# Supplementary material for: Differentiated care for youth in Zimbabwe: Outcomes across the HIV care cascade
Source: PLOS Glob Public Health. 2024 Feb 21;4(2):e0002553. doi: 10.1371/journal.pgph.0002553 (PMC10880981; doi:10.1371/journal.pgph.0002553)
Supplement: Attachment — Submitted filename: Response to Comments Document v2509.docx [file pgph.0002553.s003.docx]

Dear Jonathan Binamira Due,

Thank you for considering our manuscript for publication in Plos Global Public Health. Please see below our responses to the issues raised in blue.

We look forward to hearing from you.

Yours sincerely and on behalf of all authors,

**Chido Dziva Chikwari**

**COMMENTS TO THE AUTHOR:**

1. Where possible, we encourage authors to provide relevant data underlying their research with their submission in order to facilitate editorial and peer review, even if the data may be restricted from publication or made private by a data repository prior to article acceptance. Many repositories permit private access for review purposes, and have policies for public release at publication. For data concerning human research subjects, please anonymize the data as appropriate and follow pertinent legal and ethical protocols prior to sharing with editors and reviewers. If you are unable to share any pertinent data at the time of editorial and peer review, please kindly let the journal office know.

We have uploaded our dataset to Data Compass at LSHTM (<https://datacompass.lshtm.ac.uk>). It will be checked, and after assessment it will be uploaded on that website. We will make it freely available to download, with ID numbers removed and advise once online.

2. If you are submitting a clinical trial, it must be registered in a recognized international registry, e.g. ClinicalTrials.gov or the International Standard Randomized Controlled Trial Number (ISRCTN) Registry.

The trial is registered; however, this paper does not present the trial findings but rather data collected from the intervention and not directly linked to the trial outcomes. As such we have added the trial registration number to the abstract and submitted the published protocol as supporting information but have not added a consort checklist.

Please include the following with your submission:

1) Clinical Trials Registration Number (usually NCT or ISRCTN number) present in the Abstract section of the Manuscript File.

This has now been added to the abstract

2) CONSORT checklist or relevant reporting guideline (uploaded as supporting information)

Please see response above.

3) Trial protocol (uploaded as supporting information).

We have uploaded the published protocol paper as supporting information.

3. Please complete all items on the Human Participants Research Checklist that are relevant for your submission, by following this link: [http://journals.plos.org/plosone/s/file?id=dc11/PLOSOne_Human_Subjects_Research_Checklist.docx](http://protect-eu.mimecast.com/s/HnRxCx1g4cZX991tY9gsp?domain=journals.plos.org). There may be overlap between the checklist items and other queries listed here; please address any duplicated queries both in your response email and on the checklist itself. Upload the completed Human Participants Research checklist as file type “Other” when you re-submit your manuscript. This document is for internal journal use only and will not be published if your article is accepted. The requested information will help us to assess whether your submission complies with PLOS’s policies and adheres to applicable reporting standards. Note that your manuscript may be rejected if you provide incomplete or inadequate responses to the checklist questions and that changing the ‘Section/Category’ of your article does not affect this requirement.

We have uploaded the completed checklist as part of our manuscript resubmission. In addition, we have also uploaded the Ethical Approval Documents.
