# Supplementary material for: Differentiated care for youth in Zimbabwe: Outcomes across the HIV care cascade
Source: PLOS Glob Public Health. 2024 Feb 21;4(2):e0002553. doi: 10.1371/journal.pgph.0002553 (PMC10880981; doi:10.1371/journal.pgph.0002553)
Supplement: Attachment — Submitted filename: Response to reviewer comments v12012024.docx [file pgph.0002553.s004.docx]

Dear Jay-ar Formentera Medes,

Thank you for considering our manuscript for publication in Plos Global Public Health. Please see below our responses to the issues raised in blue.

We look forward to hearing from you.

Yours sincerely and on behalf of all authors,

**Chido Dziva Chikwari**

**COMMENTS TO THE AUTHOR:**

1. We suggest you thoroughly copyedit your manuscript for language usage, spelling, and grammar. If you do not know anyone who can help you do this, you may wish to consider employing a professional scientific editing service.

   Whilst you may use any professional scientific editing service of your choice, PLOS has partnered with both American Journal Experts (AJE) and Editage to provide discounted services to PLOS authors. Both organizations have experience helping authors meet PLOS guidelines and can provide language editing, translation, manuscript formatting, and figure formatting to ensure your manuscript meets our submission guidelines. To take advantage of our partnership with AJE, visit the AJE website ([http://learn.aje.com/plos/](http://protect-eu.mimecast.com/s/8WF6Cg204inZPrBS2jHAT?domain=learn.aje.com)) for a 15% discount off AJE services. To take advantage of our partnership with Editage, visit the Editage website ([www.editage.com](http://protect-eu.mimecast.com/s/ZwLyCj814CkQl2OH7bQc5?domain=editage.com)) and enter referral code PLOSEDIT for a 15% discount off Editage services. If the PLOS editorial team finds any language issues in text that either AJE or Editage has edited, the service provider will re-edit the text for free.

   Upon resubmission, please provide the following:

   - The name of the colleague or the details of the professional service that edited your manuscript
   - A copy of your manuscript showing your changes by either highlighting them or using track changes (uploaded as a *supporting information* file)
   - A clean copy of the edited manuscript (uploaded as the new *manuscript* file)

The manuscript has been edited by Professor Rashida Ferrand. We have provided the revised manuscript with tracked changes uploaded as supporting information as well as a clean copy of the edited manuscript.

2. We note that there were multiple versions of clean manuscript, figures and supplementary files in your submission's file inventory. Please remove the older file(s), retaining the most recent version(s) for editorial review. Please double check your submission file inventory and let us know if any files appear to be outdated or absent.

We have removed all older versions of the manuscript from the submission portal.

3. Please send a completed 'Competing Interests' statement, including any COIs declared by your co-authors. If you have no competing interests to declare, please state "The authors have declared that no competing interests exist". 

The authors have declared that no competing interests exist, these have been stated in the Competing Interests statement within the portal.

4. In the online submission form, you indicated that your data will be submitted to a repository upon acceptance. We strongly recommend all authors deposit their data before acceptance, as the process can be lengthy and hold up publication timelines. Please note that, though access restrictions are acceptable now, your entire data will need to be made freely accessible if your manuscript is accepted for publication. This policy applies to all data except where public deposition would breach compliance with the protocol approved by your research ethics board. If you are unable to adhere to our open data policy, please kindly revise your statement to explain your reasoning and we will seek the editor's input on an exemption. Please be assured that, once you have provided your new statement, the assessment of your exemption will not hold up the peer review process.

The data for this manuscript has been uploaded to the LSHTM data compass repository and can be accessed via this DOI: <https://doi.org/10.17037/DATA.00003649>
